# The Influence of the Molecular Structure of Compounds on Their Properties and the Occurrence of Chiral Smectic Phases

**DOI:** 10.3390/ma17030618

**Published:** 2024-01-27

**Authors:** Magdalena Urbańska, Monika Zając, Paweł Perkowski, Aleksandra Deptuch

**Affiliations:** 1 Institute of Chemistry, Military University of Technology, Kaliskiego 2, 00-908 Warsaw, Poland; monika.zajac@wat.edu.pl; 2 Institute of Applied Physics, Military University of Technology, Kaliskiego 2, 00-908 Warsaw, Poland; pawel.perkowski@wat.edu.pl; 3 Institute of Nuclear Physics Polish Academy of Sciences, Radzikowskiego 152, 31-342 Krakow, Poland; aleksandra.deptuch@ifj.edu.pl

**Keywords:** synthesis, ferroelectric, antiferroelectric, helical pitch, dielectric spectroscopy, X-ray diffraction

## Abstract

We have designed new chiral smectic mesogens with the -CH_2_O group near the chiral center. We synthesized two unique rod-like compounds. We determined the mesomorphic properties of these mesogens and confirmed the phase identification using dielectric spectroscopy. Depending on the length of the oligomethylene spacer (i.e., the number of methylene groups) in the achiral part of the molecules, the studied materials show different phase sequences. Moreover, the temperature ranges of the observed smectic phases are different. It can be seen that as the length of the alkyl chain increases, the liquid crystalline material shows more mesophases. Additionally, its clearing (isotropization) temperature increases. The studied compounds are compared with the structurally similar smectogens previously synthesized. The helical pitch measurements were performed using the selective reflection method. These materials can be useful and effective as chiral components and dopants in smectic mixtures targeted for optoelectronics and photonics.

## 1. Introduction

Chiral smectic liquid crystals are of great interest due to their unique properties and potential applications in various fields of science and technology [1,2,3,4,5]. Among smectics, the compounds exhibiting ferro- and antiferroelectric properties are of the most significant practical importance [6,7,8,9,10,11,12]. The ferroelectric phase (SmC*) was predicted and then discovered by Meyer et al. in 1975 [13], and antiferroelectricity in liquid crystals was discovered by Chandani et al. in 1989 [14]. Practical applications have only liquid–crystalline mixtures [15,16,17,18,19,20,21,22,23,24,25,26,27,28,29,30] because the composition’s tuning allows us to make the optimized ferro- or antiferroelectric mixtures. It is not easy to synthesize compounds with the desired properties. Formulating mixtures (based on existing compounds) that supply favorable properties, such as a wide range of the SmC* (ferroelectric) or SmC_a_* (antiferroelectric) phase, low melting point, long helical pitch, required spontaneous polarization, or a switching time, is much easier. To obtain the desired properties, the structure of liquid–crystalline molecules is designed appropriately, particularly the type and shape of the molecular core, the length of the terminal chains, the type and position of substituents, and the type of chiral center(s).

**Figure 1:**
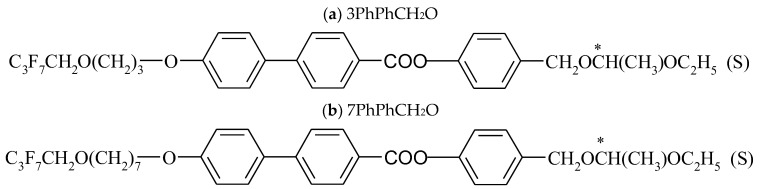
Chemical structures of new compounds with *n* = 3 (**a**) and *n* = 7 (**b**). (*) - means that the compounds are chiral.

Therefore, we designed mesogens with three benzene rings, without substituents on these rings, with a different number of methylene groups (*n* = 3 or 7), and with the chiral center: -CH_2_OC*H(CH_3_)OC_2_H_5_ (S). These mesogens are structurally like previously synthesized compounds with antiferroelectric and/or ferroelectric phases [31,32]. The chemical formulas of the designed materials are shown in Figure 1a,b. The acronym for the compounds is *n*PhPhCH_2_O.

This work aimed to synthesize and then study the mesomorphic properties of chiral smectic compounds and discuss the obtained results based on the properties of other compounds with a similar chemical structure [31,32], which were previously synthesized by the research group from the Military University of Technology. The article describes the characterizations of the mesophases obtained via polarizing optical microscopy (POM), differential scanning calorimetry (DSC), and dielectric spectroscopy. In the case of these mesogens, a rarely used –CH_2_O group was used in the chiral part of the molecule to check whether such a change would have a beneficial effect on the properties of these materials. Most often, the chiral fragment contains an ester bond (–COO–). These studies are also important for collecting information on the mesomorphic properties of the obtained mesogens and check whether they can be beneficial components for formulating liquid-crystalline mixtures.

## 2. Materials and Methods

### 2.1. Synthetic Method of Obtaining Mesogens

**Scheme 1:**
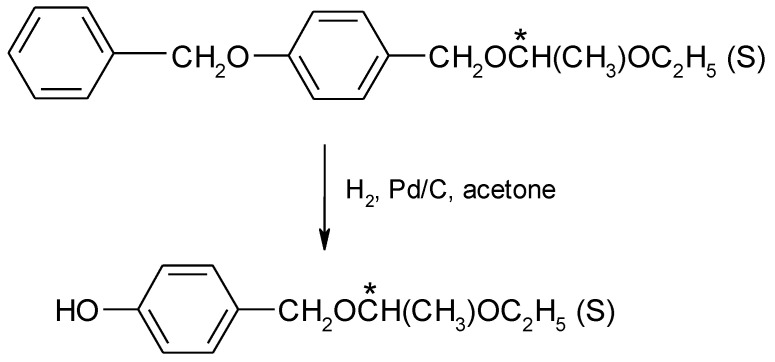
The hydrogenation reaction. (*) - means that the compounds are chiral.

The synthesis of the studied compounds was based on a two-step reaction. The benzyloxy derivative was previously synthesized by our research group according to the method described in Ref. [33]. The first stage was the hydrogenation reaction (Scheme 1), which was carried out in acetone. An amount of 2 g of catalyst was used; the temperature increased spontaneously to 29 °C during the reaction (about a liter of hydrogen was used). The whole was then heated to the boiling point, cooled, and blown out with nitrogen, and GC analysis was performed. After the reaction, the catalyst was filtered off, and acetone was distilled. The chiral phenol was crystallized from hexane (100 mL) and ethanol (20 mL). The chemical purity was checked on a Shimadzu GCMS-QP2010S series gas chromatograph (Shimadzu Co., Kyoto, Japan), which is equipped with a quadrupole mass analyzer (MS), and was 99.8%. The yield of the hydrogenation reaction was 95%. The MS yielded the following: 196(M^+^), 123, 110, 93, 81, 73, 59, 45, and 28.

**Scheme 2:**
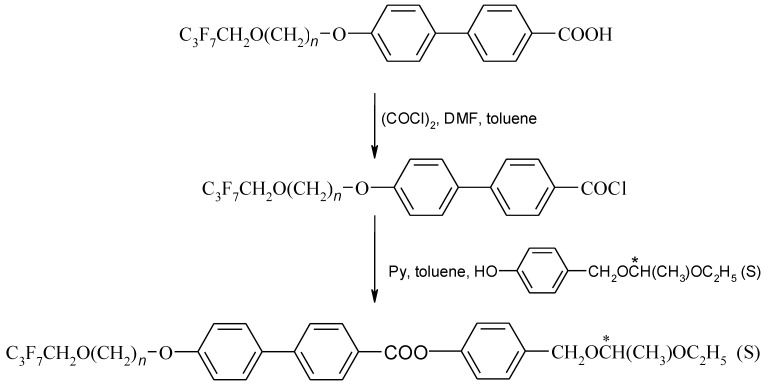
The esterification reaction. (*) - means that the compounds are chiral.

The second stage was the esterification reaction (Scheme 2), carried out in dry toluene using acid chloride with a twofold excess of pyridine. Oxalyl chloride and one drop of N,N-dimethylformamide were added to the suspension of the acid in dry toluene. There was a vigorous reaction. When the evolution of gases stopped, the mixture was heated to 30 °C with constant stirring for 5 h. The clear solution was then heated to reflux, and the excess oxalyl chloride was distilled off with the toluene on a Vigreux column. Then, phenol and pyridine were added to the cold solution. The mixture was stirred at 65 °C for 16 h, then cooled to room temperature, and poured into a solution prepared from 10% hydrochloric acid and water. The layers were separated and the organic layer was washed twice with water. The extract was filtered through activated carbon, then dried over anhydrous magnesium sulfate, and the solvent was evaporated to dryness. The obtained esters, after purification via column chromatography (silica gel and methylene chloride were used), were crystallized from anhydrous ethanol. The chemical purity of the final products was checked using thin-layer chromatography (TLC) and a high-performance liquid chromatograph, HPLC-PDA-MS (APCI-ESI dual source) Shimadzu LCMS 2010 EV (Shimadzu Co., Kyoto, Japan), which was equipped with a polychromatic UV–VIS detector (Shimadzu Co., Kyoto, Japan). The purity confirmed on the liquid chromatograph was over 99%. For ester 3PhPhCH_2_O, the MS yielded 655[M + Na]^+^, and for ester 7PhPhCH_2_O, the MS yielded 711[M + Na]^+^. The yield of the esterification reaction was below 50% in all cases. MS data for the final products are added to the Supplementary Materials in Figures S1–S2.

Proton (^1^H) and carbon (^13^C) nuclear magnetic resonance (NMR) spectra in CDCl_3_ were collected using a Bruker model AvanceIII spectrometer (Bruker, Billerica, MA, USA). All measurements were performed at room temperature. ^1^H NMR and ^13^C NMR spectra of the newly obtained mesogens are presented in Figures S3–S6 (see Supplementary Materials). NMR studies confirmed that the planned structures were obtained. The chemical shift values of the obtained compounds are given in Tables S1 and S2 in the Supplementary Materials.

### 2.2. Determination of the Enantiomeric Purity of Mesogens

A chiral stationary phase (CSP) based on cellulose was chosen as polysaccharide derivatives [34,35,36], which are the most successful CSPs for the enantioseparation of these types of mesogens. The acetonitrile/water 99/1 (*v*/*v*) mixture was suitable for enantioseparation of these enantiomers. Elution was performed in an isocratic mode. The compounds were dissolved in the mobile phase at a concentration of 0.6 mg·mL^−1^; the sample injection was 20 µL. ACN for HPLC (min. 99.9%) was purchased from POCH S.A., Gliwice, Poland. Ultrapure water was used.

**Figure 2:**
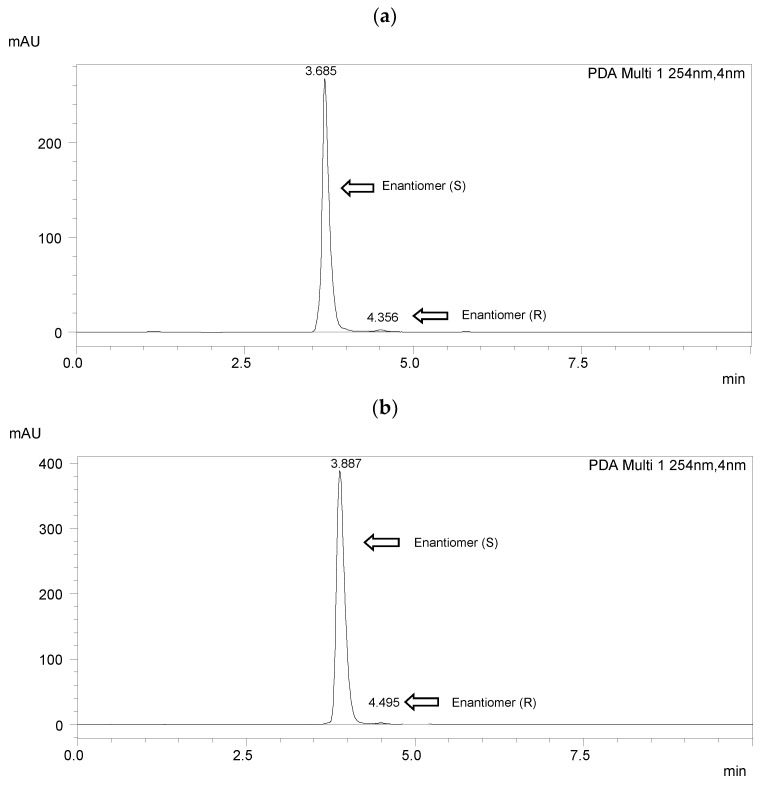
HPLC chromatograms of the enantioseparation of mesogens with labeled enantiomers. (**a**) 3PhPhCH_2_O; (**b**) 7PhPhCH_2_O.

Chiral separations were conducted using a Shimadzu LC-20AP HPLC system (Shimadzu Co., Kyoto, Japan) consisting of a binary solvent delivery pump, an autosampler (SIL-10AP), a communications bus module (CBM-20A), a diode array detector (SPD-M20A), and a fraction collector (FRC-10A). Data acquisition was performed using Shimadzu software. The measurements were conducted at room temperature. The mobile phase flow rate was 1.0 mL·min^−1^, and the detection wavelength was 254 nm. The ReproSil Chiral MIC column—cellulose tris-(3,5-dichlorophenyl-carbamate), immobilized on silica gel with a particle size of 5 μm, with dimensions of 250 mm × 4.6 mm i.d, and a pore size of 1000 Å (Dr. Maisch, Ammerbuch, Germany)—was used for the chiral separation. The enantioselective HPLC results are shown in Figure 2 and Table 1.

The developed method was successfully applied to the chiral separation of mesogens (R_s_ ˃ 2.0). The optical purity of mesogens is high, especially for mesogens with a longer oligomethylene spacer. Another round of crystallization could enantiomerically enrich these mesogens. The optical purity of the used chiral phenol was *ee* = 98.3%.

## 3. Results and Discussion

### 3.1. Transition Temperatures and Phase Behavior

The sequence of mesophases was determined via polarizing optical microscopy (POM) using an Olympus BX51 polarizing microscope (Shinjuku, Tokyo, Japan) equipped with a Linkam heating/cooling stage THMS-600 (Linkam Scientific Instruments Ltd., Tadworth, UK). A differential scanning calorimetry DSC 204 F1 Phoenix instrument (Netzsch, Selb, Germany) was used to estimate the enthalpies and temperatures of phase transitions. In both cases, observations were carried out in a heating and cooling cycle near the phase transition region at a scan rate of 2 °C·min^−1^. The phase transition temperatures and the phase assignments made based on these observations are given in Table 2.

**Figure 3:**
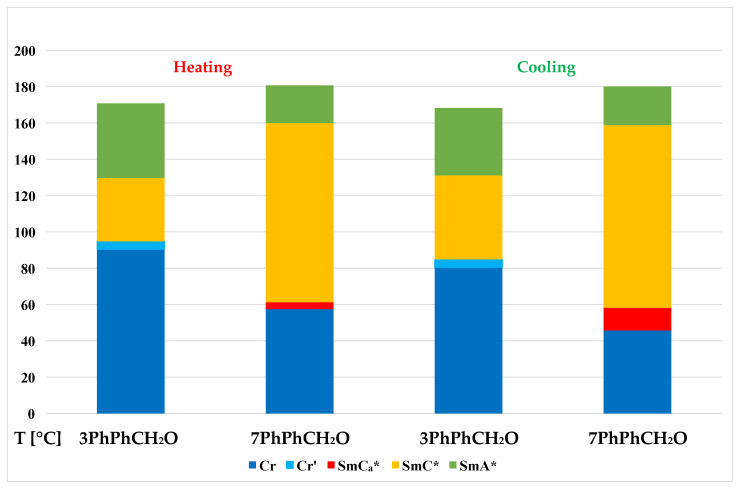
Phase diagram for new mesogens (DSC measurements in the heating and the cooling cycles).

Figure 3 shows the phase temperature ranges for obtained mesogens to visualize the mesomorphic behavior better. The y-axis shows the phase transition temperatures in [°C], and the x-axis shows the acronyms of the compounds (this applies to all phase diagrams).

**Figure 4:**
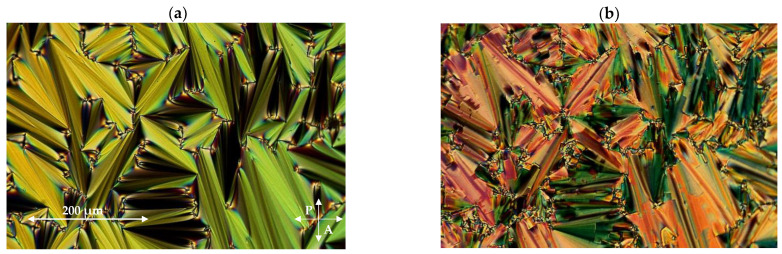

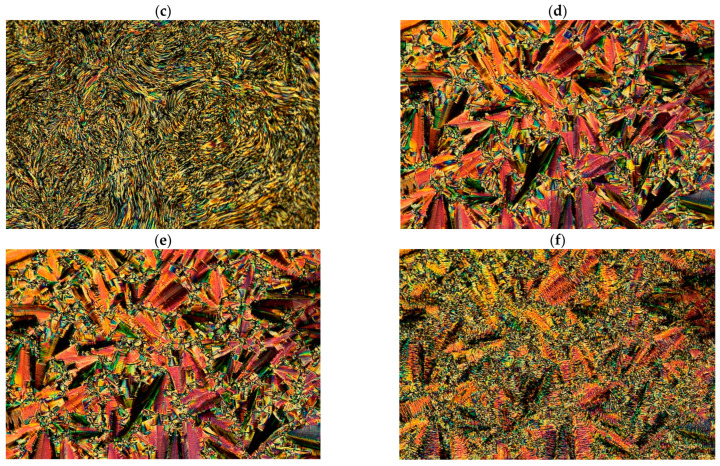
Microphotographs of the characteristic POM textures obtained for different mesophases for the compound 3PhPhCH_2_O, including (**a**) SmA* at T = 165.7 °C, (**b**) SmC* at T = 136.3 °C, and (**c**) the Cr phase at T = 69.7 °C, and for the compound 7PhPhCH_2_O: (**d**) SmC* at T = 68.5 °C; (**e**) SmC_a_* at T = 56.6 °C; (**f**) the Cr phase at T = 40.5 °C. All textures were observed during the cooling cycle.

Both studied compounds have the SmC* and SmA* phases. The ferroelectric phase exists in a very wide temperature range for the compound with the longer oligomethylene spacer (about 100 °C). For the compound 3PhPhCH_2_O, this phase occurs in a narrower temperature range (about 40 °C). The smectic A* phase occurs in a wide (for the compound with *n* = 3) or medium (for the compound with *n* = 7) temperature range. The antiferroelectric phase (SmC_a_*) occurs only for the compound 7PhPhCH_2_O in a very narrow (about 4 °C) temperature range in the heating cycle. Upon cooling, the range of the SmC_a_* phase becomes wider (about 12 °C). The compound with the shorter oligomethylene spacer has a lower clearing point and, at the same time, has a higher melting point. Microphotographs of the characteristic textures obtained under crossed polarizers in POM for mesogens are shown in Figure 4.

**Figure 5:**
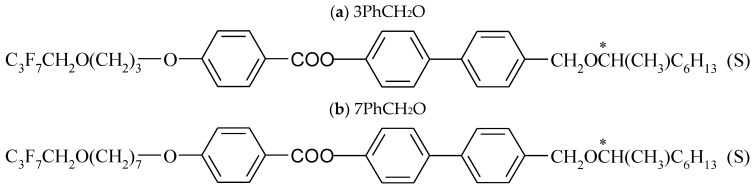
Chemical structures of previously synthesized compounds with *n* = 3 (**a**) and *n* = 7 (**b**). (*) - means that the compounds are chiral.

**Figure 6:**
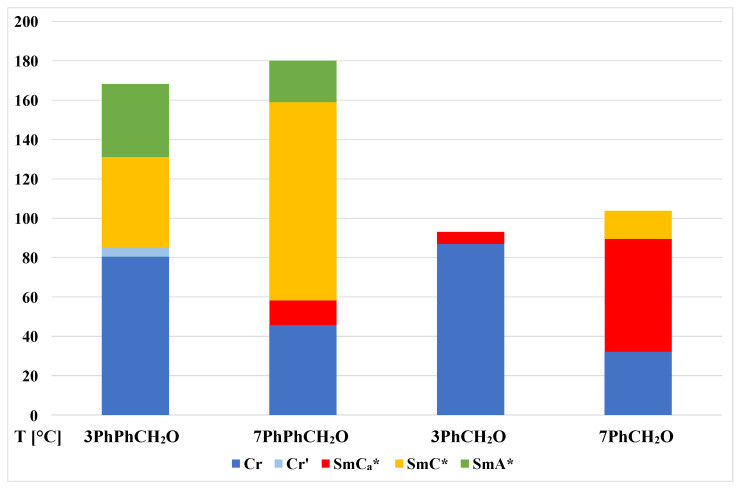
Phase transition temperatures for the compounds with the acronyms 3PhCH_2_O, 7PhCH_2_O, 3PhPhCH_2_O, and 7PhPhCH_2_O observed in the cooling cycle.

Figure 5 shows the formulas and acronyms of compounds that were used to compare phase temperatures with the compounds obtained in this work. We have different phase situations for the structurally similar compounds [31,32] to those described above; see Figure 6. The main differences between the compounds presented in this work and the compounds described earlier are the structure of the rigid core (PhPhCOOPh or PhCOOPhPh) and different chains creating the chiral fragment (–C_6_H_13_ or –OC_2_H_5_).

First, there are very large differences in the clearing points for the compared compounds. For the compound 3PhCH_2_O, only the monotropic SmC_a_* phase is observed in a narrow temperature range. The compound 7PhCH_2_O has this phase in a wide temperature range and the ferroelectric phase in a narrow range. Figure 6 shows how the change in the order of occurrence of benzene rings and different chiral centers affects the mesomorphic properties. The compounds studied in this work exhibit primarily ferroelectric properties, while those synthesized earlier have antiferroelectric properties. It can also be seen that compounds with longer terminal chains have wider ranges of these phases.

**Figure 7:**
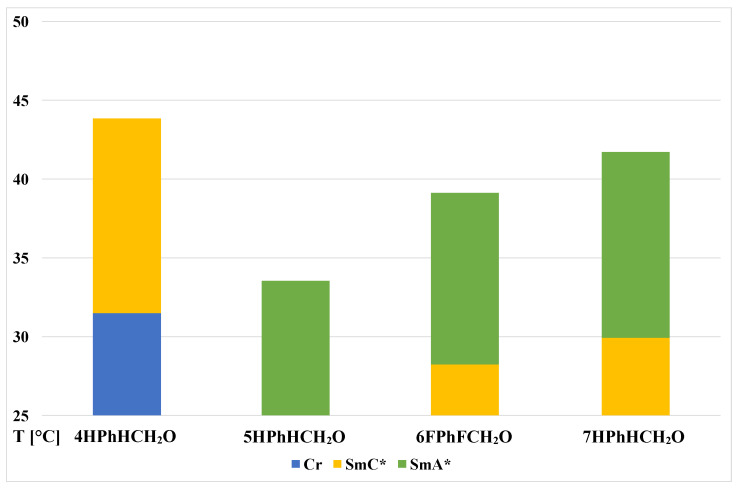
Phase transition temperatures for two-ring compounds observed in the cooling cycle.

**Figure 8:**
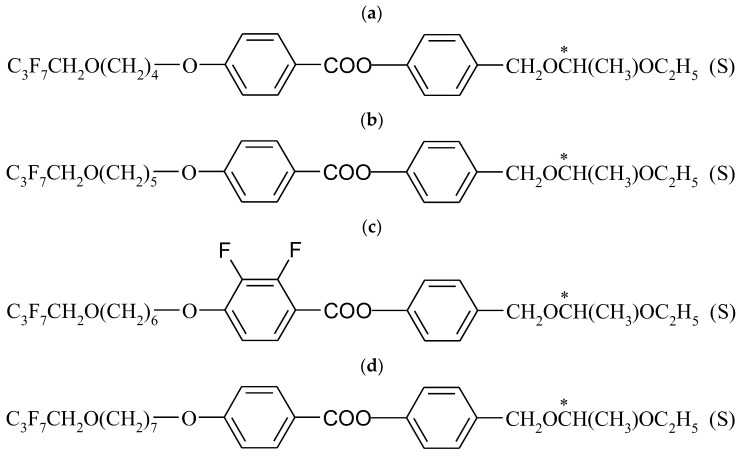
Chemical structures of two-ring compounds with different numbers of oligomethylene spacers. (**a**) 4HPhHCH_2_O; (**b**) 5HPhHCH_2_O; (**c**) 6FPhFCH_2_O; (**d**) 7HPhHCH_2_O. (*) - means that the compounds are chiral.

How the number of benzene rings affects the mesomorphic properties is shown in Figure 7, because several two-ring compounds with the same chiral center as that of the studied mesogens, –CH_2_OC*H(CH_3_)OC_2_H_5_ (S) were also synthesized. Figure 8 shows the chemical structures of these materials.

Two-ring compounds have much lower clearing points than three-ring compounds, which are below 45 °C. One compound (with *n* = 4) has the ferroelectric phase, one (with *n* = 5) has the SmA* phase, and the two compounds (with the longest oligomethylene spacer) have both of these phases. These compounds also have very low melting points. There is a significant difference in the phase temperature ranges between compounds differing in the number of benzene rings.

### 3.2. Dielectric Spectroscopy and Spontaneous Polarization

Impedance spectroscopy is a useful method for characterizing the dielectric properties of liquid–crystalline materials. In our laboratory, we have an impedance analyzer from Hewlett Packard: HP 4192A. This equipment allows us to nominally perform measurements at frequencies from 5 Hz to 13 MHz. We used the measurement range from 100 Hz to 10 MHz. For measurements, we used self-made cells with gold electrodes instead of cells with ITO electrodes to avoid high-frequency parasitic effects [37]. Such cells can be used for frequencies up to 10 MHz. The thickness of the cells used was about 5 µm, and the alignment of the cells was planar (polyimide SE130). The liquid crystals were heated and placed in the measurement cell in the isotropic phase using capillary action. All measurements were performed in the cooling cycles. We performed measurements without a DC field or with a 10 V DC field.

The dielectric spectroscopy measurement confirmed the phase sequence for the studied mesogens. All measurements were performed at a low cooling rate of 0.5 °C/min. 

**Figure 9:**
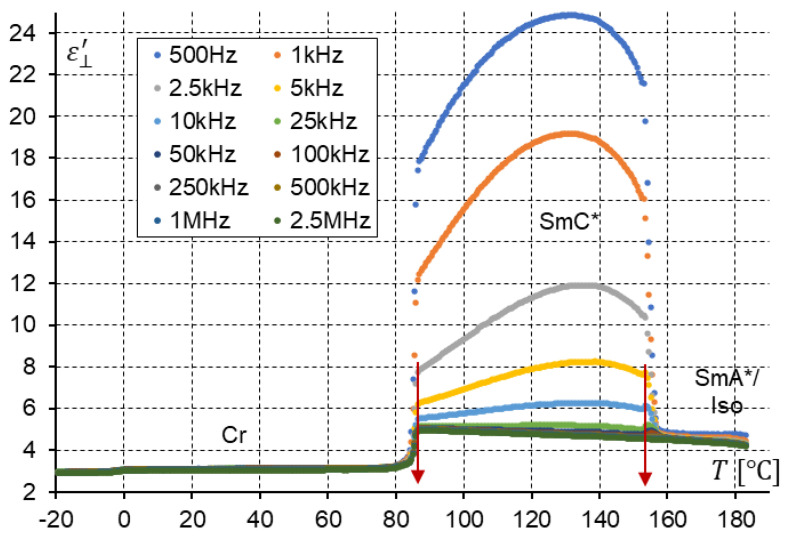
The real part, $\varepsilon_{⊥}^{’}$, of dielectric permittivity for the compound 3PhPhCH_2_O versus temperature at twelve frequencies, measured at cooling without a DC field.

**Figure 10:**
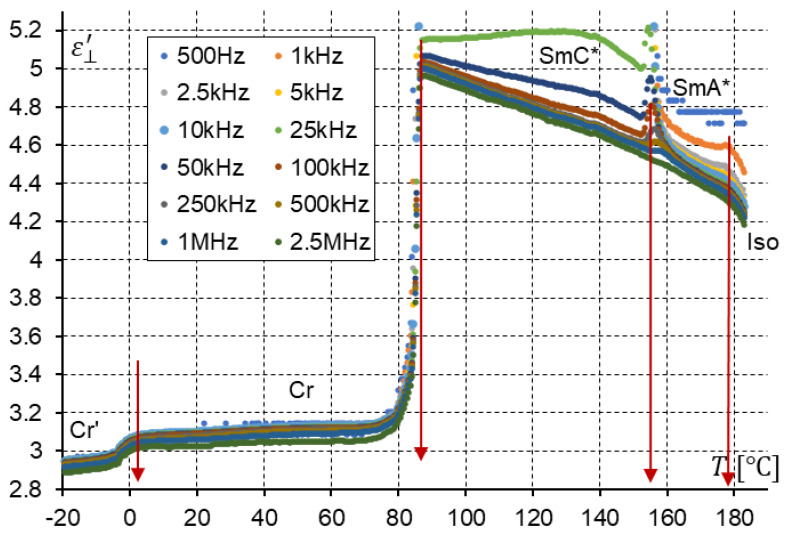
The real part, $\varepsilon_{⊥}^{’}$, of dielectric permittivity for the compound 3PhPhCH_2_O versus temperature at twelve frequencies, measured at cooling without a DC field.

The compound 3PhPhCH_2_O was cooled from the isotropic liquid (183 °C) to the molecular crystal (−20 °C). Figure 9 shows the real part of dielectric permittivity versus temperature, measured for twelve frequencies. This plot suggests that around 154 °C, the ferroelectric phase (SmC*) is created, while below 87 °C, a molecular crystal nucleates. A strong dielectric response related to the Goldstone mode confirms this phase. To find out more, we changed the scale for the vertical axis. The result is presented in Figure 10.

When we change the scale of the $\varepsilon_{⊥}^{’}\;$axis, two additional phases are visible: the SmA* phase and the second crystalline phase (Cr’). The SmA* phase nucleates from isotropic liquid at 179 °C and transforms into the SmC* phase, while the Cr’ phase nucleates from the Cr phase at 3 °C. It seems that no dispersion is detected in both crystalline phases (Cr and Cr′). 

**Figure 11:**
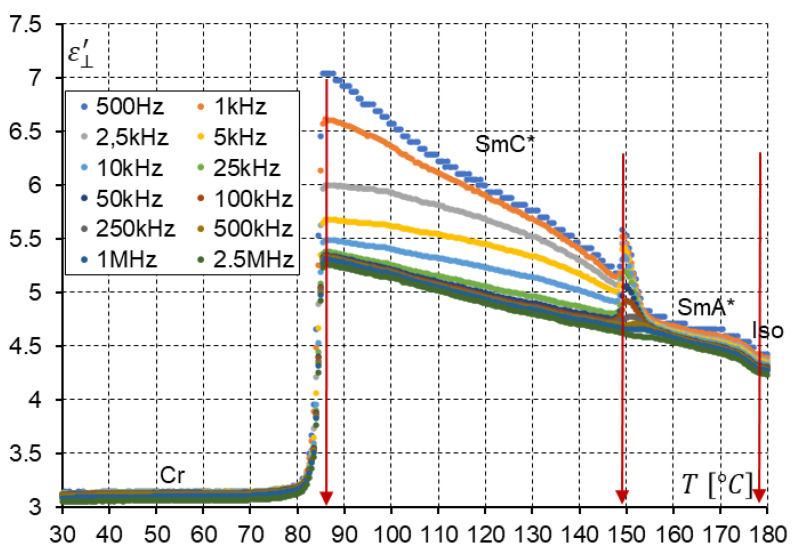
The real part, $\varepsilon_{⊥}^{’}$, of dielectric permittivity for the compound 3PhPhCH_2_O versus temperature for twelve frequencies, measured at cooling with the 10 V DC field.

To be sure that the SmA* phase precedes the SmC* phase, an additional measurement at cooling, with a 10 V DC field, was performed. The results are shown in Figure 11. The DC field partially suppresses the Goldstone mode. At the border in the SmA*–SmC* phase transition, the weak but clear soft mode is detected. In measurements performed without a DC field, the soft mode was covered by the strong Goldstone mode. Knowing these experimental results, we can specify the observed phases with the temperatures of the phase transition at cooling: Iso 179 °C; SmA* 154 °C; SmC* 87 °C; Cr 3 °C Cr’. Additional 3D plots showing the dielectric properties of the compound 3PhPhCH_2_O are presented in the Supplementary Materials in Figures S7–S8. 

**Figure 12:**
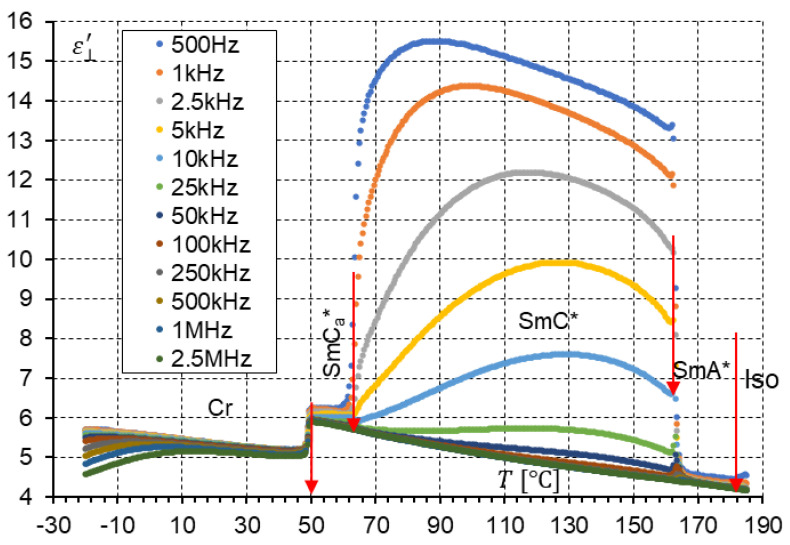
The real part, $\varepsilon_{⊥}^{’}$, of dielectric permittivity for the compound 7PhPhCH_2_O versus temperature for twelve frequencies measured at cooling without a DC field.

The compound 7PhPhCH_2_O was cooled from the isotropic liquid (187 °C) to a molecular crystal (−30 °C). Figure 12 shows the real part of dielectric permittivity versus temperature, which is measured for twelve frequencies. This plot suggests that around 163.5 °C, the ferroelectric phase (SmC*) is created. SmC* gradually changes, reaching the SmC_a_* phase at 63.5 °C. This smectic phase is crystallized at 50 °C. Interestingly, the phase below SmC_a_* exhibits one high-frequency relaxation.

**Figure 13:**
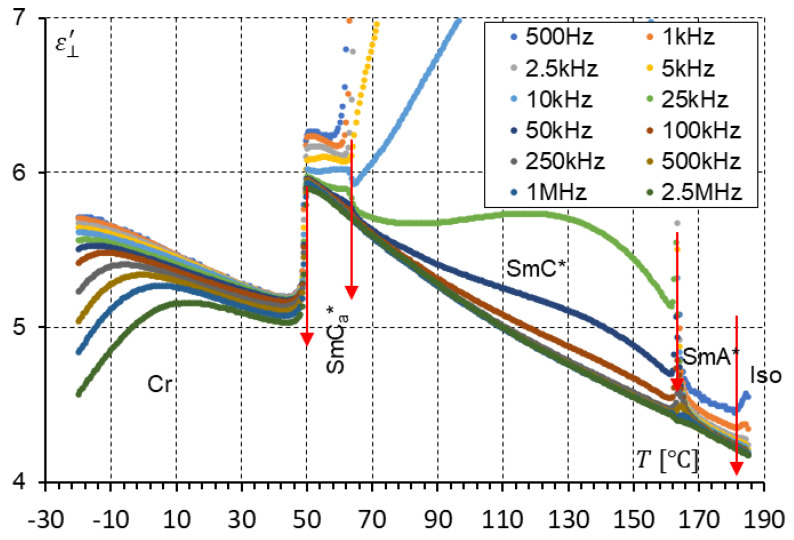
The real part, $\varepsilon_{⊥}^{’}$, of dielectric permittivity for the compound 7PhPhCH_2_O versus temperature for twelve frequencies measured at cooling without a DC field.

The vertical axis was changed to see more details, and new plots are seen in Figure 13. This figure shows that the SmA* phase nucleates from isotropic liquid at 182 °C. Additionally, in Figure 13, the SmA* (soft mode), SmC_a_*, and Cr dispersion is clearly visible.

**Figure 14:**
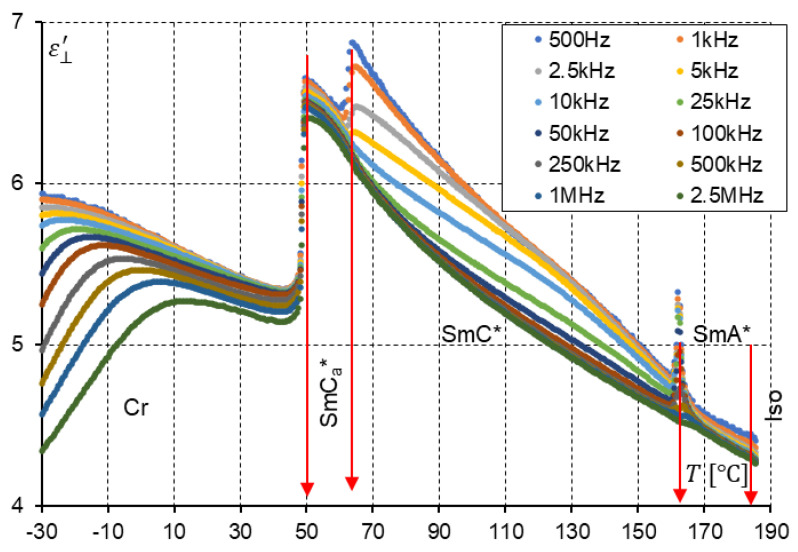
The real part, $\varepsilon_{⊥}^{’}$, of dielectric permittivity for the compound 7PhPhCH_2_O versus temperature for twelve frequencies, measured at cooling with a 10 V DC field.

To confirm the SmC* and SmA* phases, a measurement at cooling was performed with a 10 V DC field applied (see Figure 14). We see that the Goldstone mode is almost suppressed (as is observed for the compound 3PhPhCH_2_O). The soft mode is also detectable, confirming the SmA*–SmC* phase transition. Notably, a 10 V DC field is enough to suppress the dispersion in the SmC_a_*. The DC field unwinds the helicoidal structure in the SmC_a_*, and both collective modes, P_L_ and P_H_, are suppressed, as was earlier found by our group [38,39]. The molecular S-mode still exists in SmC_a_* (it is not fully suppressed by the DC field). The DC field slightly increases the electric response of the SmC_a_* and Cr phases.

Knowing these experimental results, we can specify the observed phases with the temperatures of the phase transition at cooling: Iso 182 °C; SmA* 154 °C; SmC* 63.5 °C; SmC_a_* 50 °C Cr. Additional 3D plots showing the dielectric properties of the compound 7PhPhCH_2_O are presented in the Supplementary Materials in Figures S9–S10.

**Figure 15:**
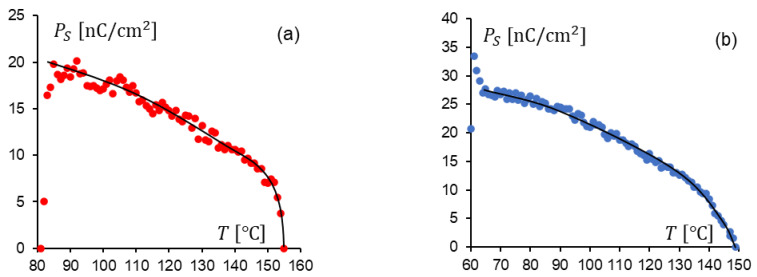
The spontaneous polarization versus temperature for the compounds 3PhPhCH_2_O (**a**) and 7PhPhCH_2_O (**b**).

To confirm that observed phases are polar (ferro- or antiferroelectric), additionally, spontaneous polarization, *P_S_*, was measured (Figure 15). We used the standard reversal current method. We integrated it into the time domain of the polarization peak. The spontaneous polarization was rather low, but the polarization peak was clearly visible in our experiment for both compounds. For measurements, we used a 5 µm thick cell planarly aligned with the gold electrodes. The external field was 50 Hz and 20 Vpp. We are afraid the results for low temperatures (60–63 °C) for the compound 7PhPhCH_2_O are not correct because the current peak was not well defined. The temperature range of the existence of spontaneous polarization in the compound 7PhPhCH_2_O is shifted a little to the left in comparison with the results shown in Figure 12 and Figure 13.

### 3.3. X-ray Diffraction

**Figure 16:**
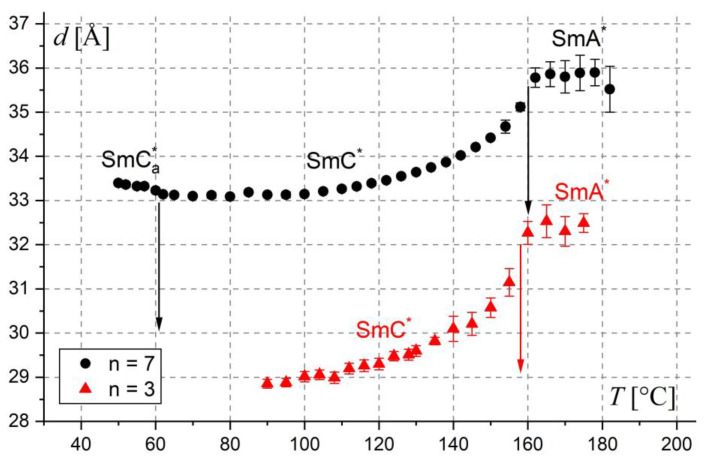
Smectic layer spacing of the compounds nPhPhCH_2_O (*n* = 3, 7) as a function of temperature.

The smectic layer spacing (Figure 16) was determined via the X-ray diffraction method [40], using the X’Pert PRO diffractometer (Malvern PANalytical, Malvern, Worcestershire, UK) with a TTK-450 temperature attachment (Anton Paar, Graz, Austria). Before the measurement, the samples were heated above the clearing temperature, and afterward, the diffraction patterns in the 2θ = 1.7–30° range (CuKα radiation, Bragg-Brentano geometry) were collected upon cooling. Data analysis was performed using WinPLOTR [41] and OriginPro.

The layer spacing in the SmA* phase is constant within uncertainties, while in the SmC* phase, it decreases with decreasing temperature. In the SmC_a_* phase of the compound 7PhPhCH_2_O, the layer spacing is slightly larger than in the SmC* phase, which enables observation of this transition. The layer shrinkage at the SmA*/SmC* transition is only ca. 3.5% for the compound 3PhPhCH_2_O and 2% for the compound 7PhPhCH_2_O. It suggests the possible presence of the SmA* phase of the de Vries type [42], where the molecules are already tilted in random directions and only the average tilt angle is equal to zero. The border layer shrinkage dividing the de Vries SmA*/SmC* and conventional SmA*/SmC* transitions is 1–5%, according to various authors [43,44,45]. Compounds with a small layer shrinkage are characterized by a smaller number of defects in alignment after the transition to the tilted smectic phase. Therefore, they are better for practical use in displays than the compounds with a more significant layer shrinkage [44,45].

### 3.4. Helical Pitch Measurements

The helical pitch length measurements were performed according to the detailed procedure described in Ref. [22] using a Shimadzu UV–Vis–NIR spectrophotometer (UV-3600, Shimadzu Co., Kyoto, Japan). The spectrophotometer is equipped with a temperature controller, U7 MLW, with a Peltier element. Before measurements, a thin layer of orienting surfactant was applied to the glass plate to force the required homeotropic alignment of the liquid-crystalline molecules. After baseline collection, liquid–crystalline samples were applied on the surface of the slide, and the wavelength of selectively reflected light was recorded. The measurements were performed on the cooling cycle.

For both compounds, the wavelength of selective reflection was above the measuring range of the spectrophotometer (360–3000 nm). On this basis, we can conclude that the helical pitch is exceptionally long, which has already been observed previously for the compounds with the –CH_2_O group near the chiral center [31].

## 4. Conclusions

The synthesized materials show the following phase sequence: Cr′–Cr–SmC*–SmA*–Iso or Cr–SmC_a_*–SmC*–SmA*–Iso depending on the length of the terminal achiral chain. The chiral center, quite rarely used with the –CH_2_O group, is also important for mesomorphic properties. The mesogens exhibit primarily ferroelectric properties. The antiferroelectric phase is observed for one of these compounds in a narrow temperature range. The dielectric spectroscopy used in this work fully confirms the identification of these mesophases. The smectic liquid crystals designed and studied in this work are characterized by good chemical stability, high optical purity, and appropriate physical properties. Therefore, they can be useful as chiral components in antiferroelectric and ferroelectric mixtures. The formulation of such mixtures is in progress, and their physicochemical and electro-optical properties will be presented in the next article. These types of chiral materials can be used in nonlinear optics, LC displays, LC-based smart windows, optical storage devices, light-emitting transistors, and 3D imaging technologies, among others [46,47,48,49,50,51,52,53,54,55,56].

The obtained results make a significant contribution to a better understanding of the relationship between the structure of the molecule and the occurrence of chiral smectic phases.

## Figures and Tables

**Table 1:** Relative peak areas and resolution of chiral mesogens obtained from chiral HPLC analysis and corresponding enantiomeric excess values of the enantiomers.

Peak Area (%)	3PhPhCH_2_O	7PhPhCH_2_O
**(S)**	99.246	99.640
**(R)**	0.754	0.360
**% *ee***	98.5	99.3
**R_s_**	2.24	2.03

**Table 2:** Thermal properties of mesogens.

Acronym	Phase Transition Behavior ^a^								
7PhPhCH_2_O	**Cr**	51.6–58.6	SmC_a_*	60.7–63.1	SmC*	158.2–162.8	SmA*	179.1–183.6	**Iso**
		44.2–45.8		57.3–59.4		152.1–159.3		175.1–182.4	
		57.5		61.4		160.1		180.7	
		45.7		58.3		159.0		180.1	
		*22.4*		*0.05*		*0.18*		*6.7*	
3PhPhCH_2_O	**Cr**	90.4–93.5	-		SmC*	149.5–152.5	SmA*	168.3–175.9	**Iso**
		74.7–77.6				143.4–148.6		168.2–171.1	
		90.4; 94.9				129.8		169.0	
		80.5; 85.0				131.1		168.2	
		*15.1; 1.7*				*0.30*		*4.6*	

^a^ The first row contains POM measurements in the heating cycle (°C); the second row contains POM measurements in the cooling cycle (°C); the third row contains DSC measurements in the heating cycle (°C); the fourth row contains DSC measurements in the cooling cycle (°C); the fifth row contains enthalpies (kJ·mol^−1^). (*) - means that the compounds are chiral.

## Data Availability

The data presented in this study are available on request from the corresponding author.

## Supplementary Materials

The following supporting information can be downloaded at https://www.mdpi.com/article/10.3390/ma17030618/s1: Figures S1–S10; Tables S1 and S2.
