# Supplementary material for: The Influence of the Molecular Structure of Compounds on Their Properties and the Occurrence of Chiral Smectic Phases
Source: Materials (Basel). 2024 Jan 27;17(3):618. doi: 10.3390/ma17030618 (PMC10856103; doi:10.3390/ma17030618)
Supplement: Supplementary file 1 [file materials-17-00618-s001.zip › materials-2807500-supplementary.pdf]

Supplementary materials for the article

The influence of the molecular structure of compounds on their properties and the occurrence of chiral smectic phases

Magdalena Urbańska[1*], Monika Zając[1], Paweł Perkowski[2], Aleksandra Deptuch[3]

[1]*Institute of Chemistry, Military University of Technology, Kaliskiego 2, 00-908 Warsaw, Poland*

[2]*Institute of Applied Physics, Military University of Technology, Kaliskiego 2, 00-908 Warsaw, Poland*

[3]*Institute of Nuclear Physics Polish Academy of Sciences, Radzikowskiego 152, 313-42 Krakow, Poland*

**Corresponding author: Magdalena Urbańska, e-mail: magdalena.urbanska@wat.edu.pl*

MASS SPECTRA OF MESOGENS

The purity of the liquid crystalline esters was recorded using a Shimadzu prominence chromatograph. The strong molecular ion with a captured sodium atom $[M + Na]^+$ was observed; see Figures S1 and S2.

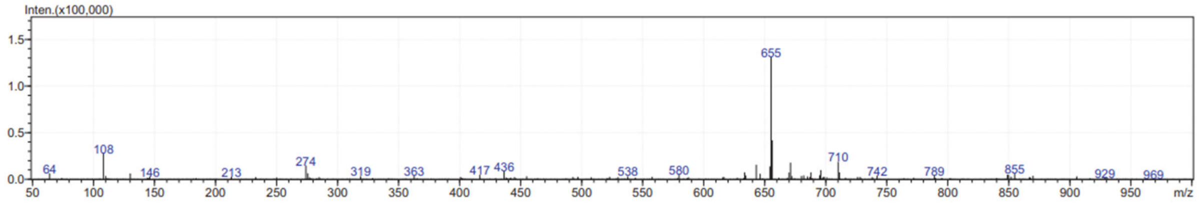

Figure **S1.** Mass spectrum of the compound $3PhPhCH_2O$.

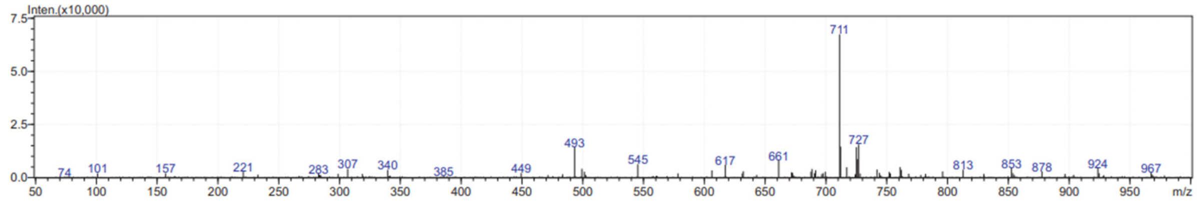

Figure S2. Mass spectrum of the compound 7PhPhCH$_2$O.

STRUCTURE CONFIRMATION OF MESOGENS

A comparison of NMR spectra confirmed the compliance of real structures with the planned structures; see Figures S3-S6.

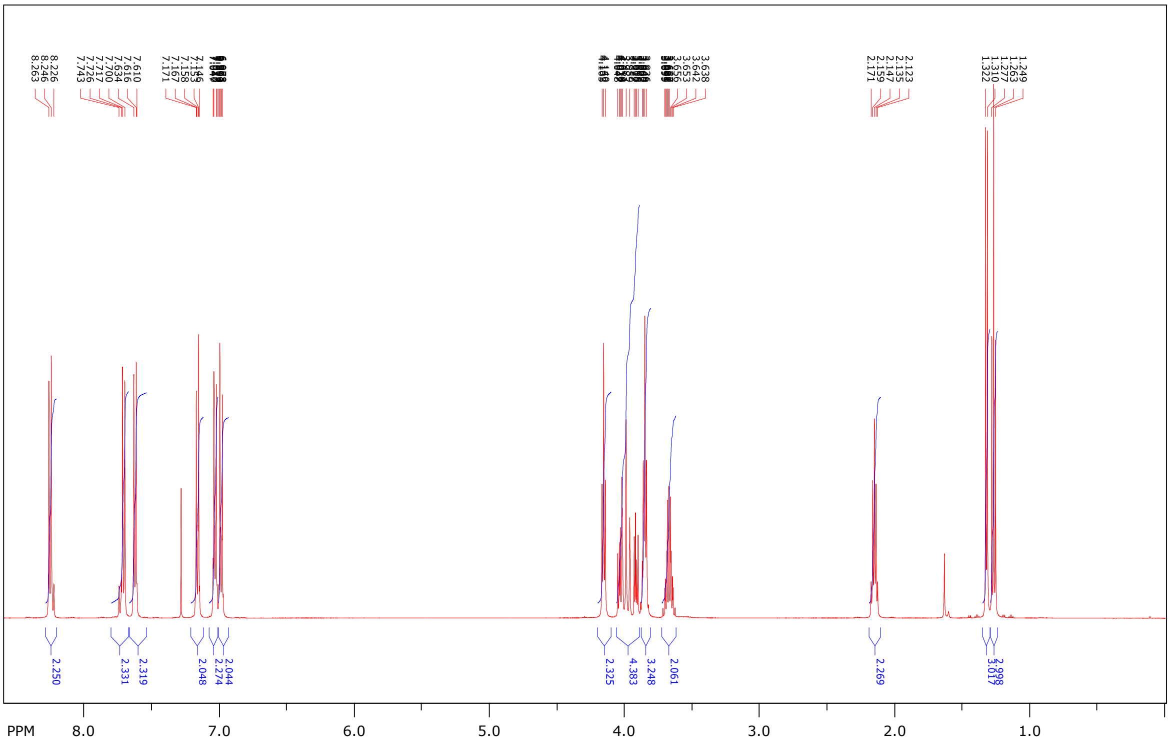

Figure S3. ^{1}H NMR spectrum of the compound 3PhPhCH$_2$O in CDCl$_3$.

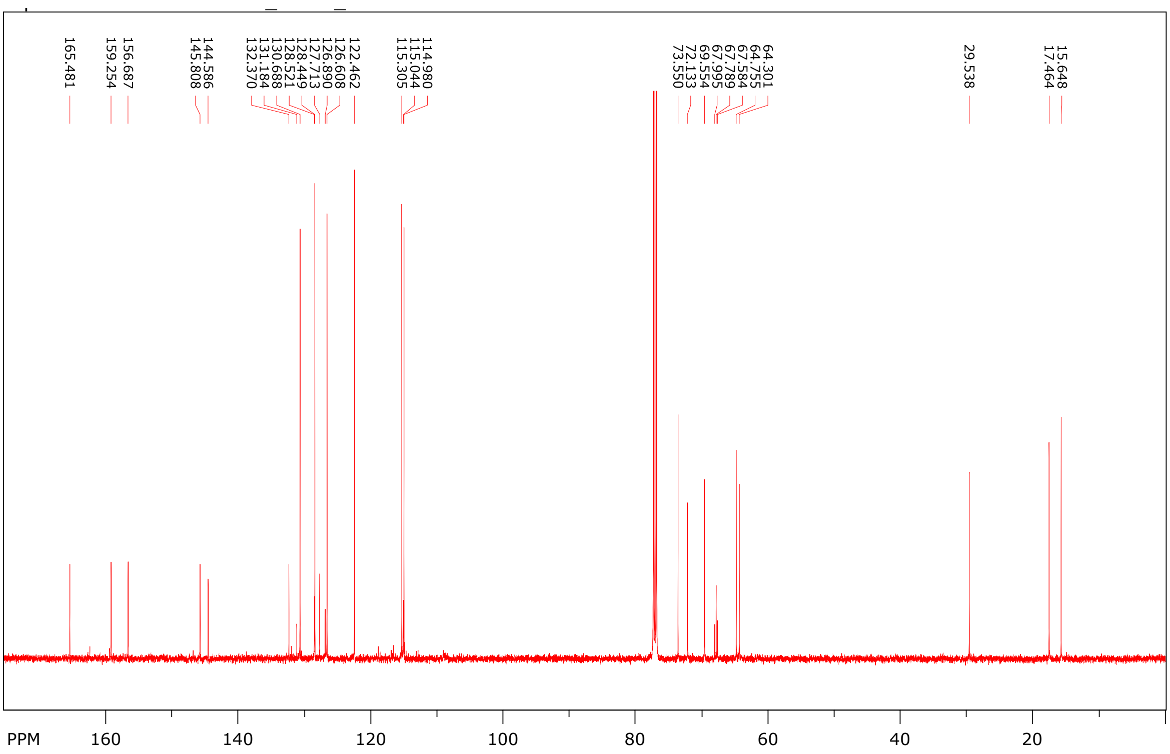

Figure S4. ^{13}C NMR spectrum of the compound 3PhPhCH$_2$O in CDCl$_3$.

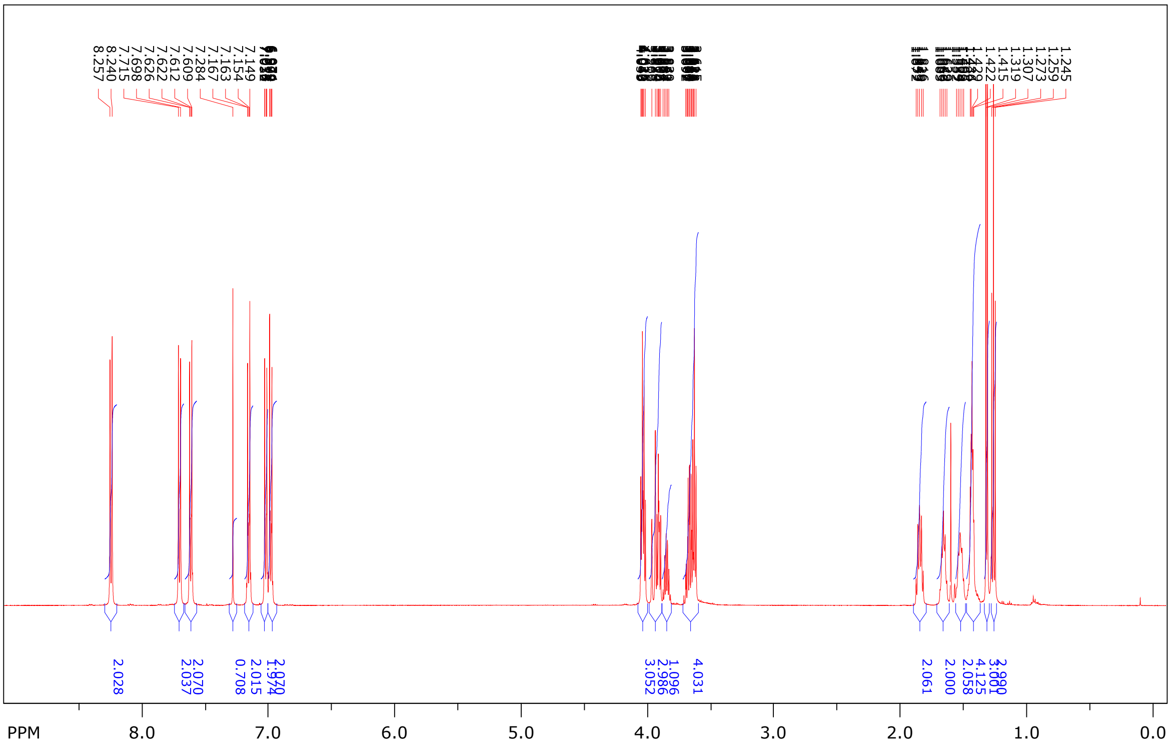

Figure S5. ^{1}H NMR spectrum of the compound 7PhPhCH$_2$O in CDCl$_3$.

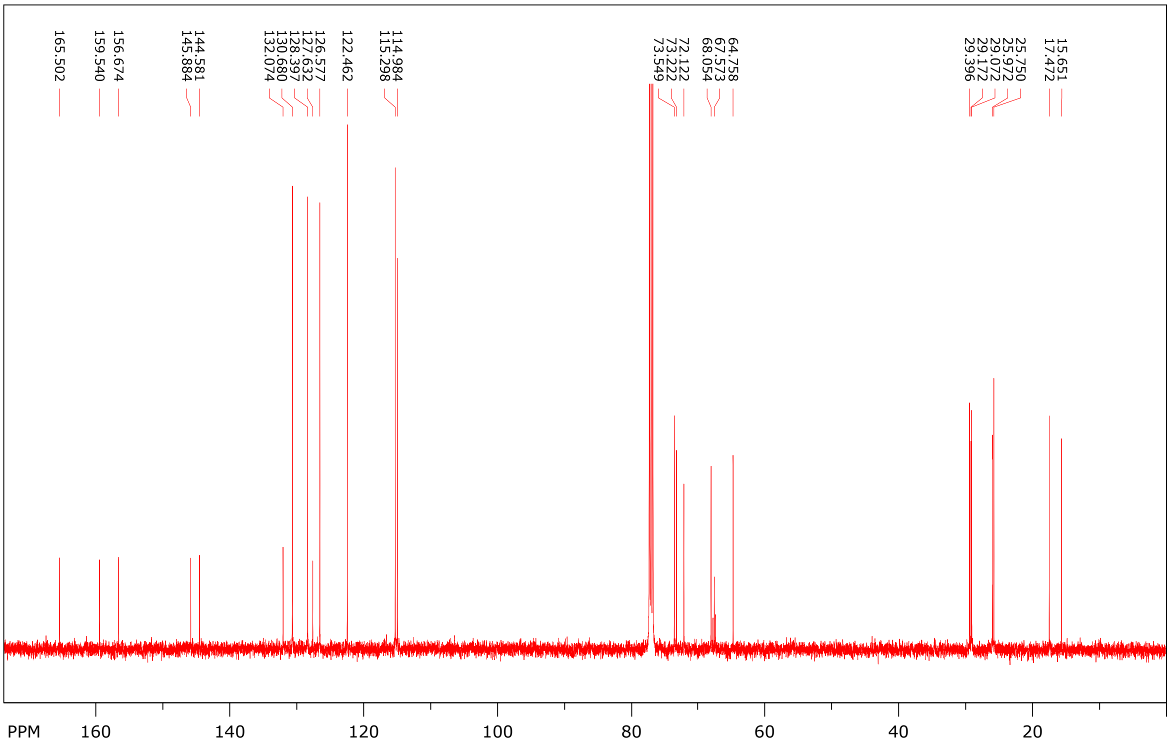

Figure S6. ^{13}C NMR spectrum of the compound 7PhPhCH$_2$O in $CDCl_3$.

The chemical shift values for obtained mesogens are given in Tables S1 and S2. Protons are marked as shown below:

A. 3PhPhCH$_2$O

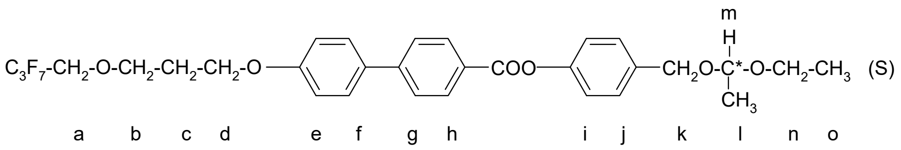

B. 7PhPhCH$_2$O

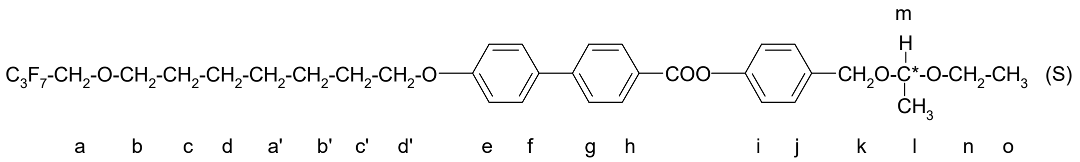

Table S1. ^{1}H NMR chemical shift data (ppm) for mesogens in $CDCl_3$ solution.

<table>
<tr><th>Protons</th><th>Mesogen A.</th><th>Mesogen B.</th></tr>
<tr><td>a</td><td>3.917 (2H, t)</td><td>3.914 (2H, t)</td></tr>
<tr><td>b</td><td>3.670 (2H, t)</td><td>3.664 (2H, t)</td></tr>
<tr><td>c</td><td>2.147 (2H, m)</td><td rowspan="5">1.415-1.872 (10H, m)</td></tr>
<tr><td>d</td><td>4.037 (2H, t)</td></tr>
<tr><td>a′</td><td></td></tr>
<tr><td>b′</td><td></td></tr>
<tr><td>c′</td><td></td></tr>
<tr><td>d′</td><td></td><td>3.966 (2H, t)</td></tr>
<tr><td>e</td><td>7.023 (2H, d)</td><td>7.028 (2H, d)</td></tr>
<tr><td>f</td><td>7.158 (2H, d)</td><td>7.163 (2H, d)</td></tr>
<tr><td>g</td><td>7.616 (2H, d)</td><td>7.622 (2H, d)</td></tr>
<tr><td>h</td><td>7.726 (2H, d)</td><td>7.715 (2H, d)</td></tr>
<tr><td>i</td><td>6.992 (2H, d)</td><td>6.988 (2H, d)</td></tr>
<tr><td>j</td><td>8.246 (2H, d)</td><td>8.257 (2H, d)</td></tr>
<tr><td>k</td><td>3.860 (2H, t)</td><td>3.853 (2H, t)</td></tr>
<tr><td>l</td><td>1.310 (3H, d)</td><td>1.307 (3H, d)</td></tr>
<tr><td>m</td><td>4.152 (1H, m)</td><td>4.040 (1H, m)</td></tr>
<tr><td>n</td><td>1.322 (2H, m)</td><td>1.319 (2H, m)</td></tr>
<tr><td>o</td><td>1.263 (3H, t)</td><td>1.259 (3H, t)</td></tr>
</table>

Table S2. Values of chemical shifts for the chiral center (atoms "m") of mesogens in ^{1}H and ^{13}C NMR spectra.

**Mesogen A.**	4.152<br>67.789	^{1}H NMR [ppm]<br>^{13}C NMR [ppm]
**Mesogen B.**	4.040<br>68.054	^{1}H NMR [ppm]<br>^{13}C NMR [ppm]

3D PLOTS OF IMAGINARY PART $\varepsilon''_{\perp}$ OF DIELECTRIC PERMITTIVITY VERSUS FREQUENCY AND TEMPERATURE AT COOLING WITH THE 10 V DC FIELD FOR BOTH STUDIED MESOGENS

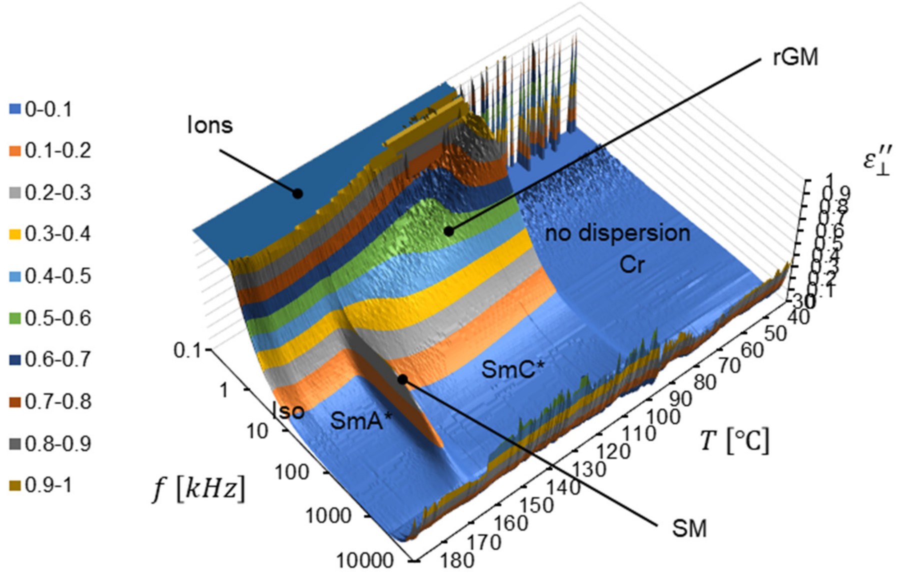

Figure S7. Imaginary part $\varepsilon''_{\perp}$ of dielectric permittivity for the compound 3PhPhCH$_2$O. In 5 μm thin cell measurement at cooling, planarly aligned, with gold electrodes. Cooling rate 0.5°C/min. The 10 V DC field. Isotropic liquid, the SmA* (soft mode: SM) and the SmC* (residual Goldstone mode: rGM) phases, and molecular crystal (Cr) are observed.

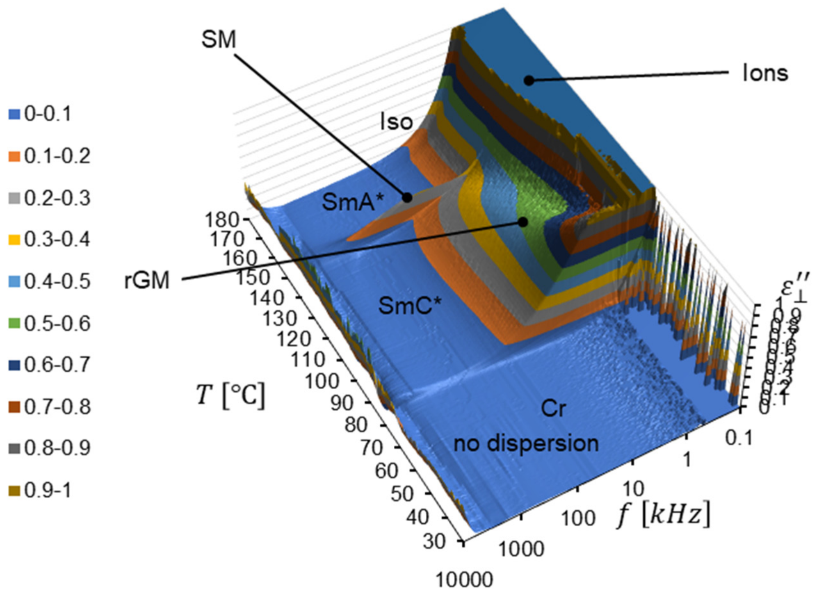

Figure S8. Imaginary part $\varepsilon''_{\perp}$ of dielectric permittivity for the compound 3PhPhCH2O. In 5 μm thin cell measurement at cooling, planarly aligned, with gold electrodes. Cooling rate 0.5°C/min. The 10 V DC field. Isotropic liquid, the SmA* (soft mode: SM) and the SmC* (residual Goldstone mode: rGM) phases, and molecular crystal (Cr) are observed.

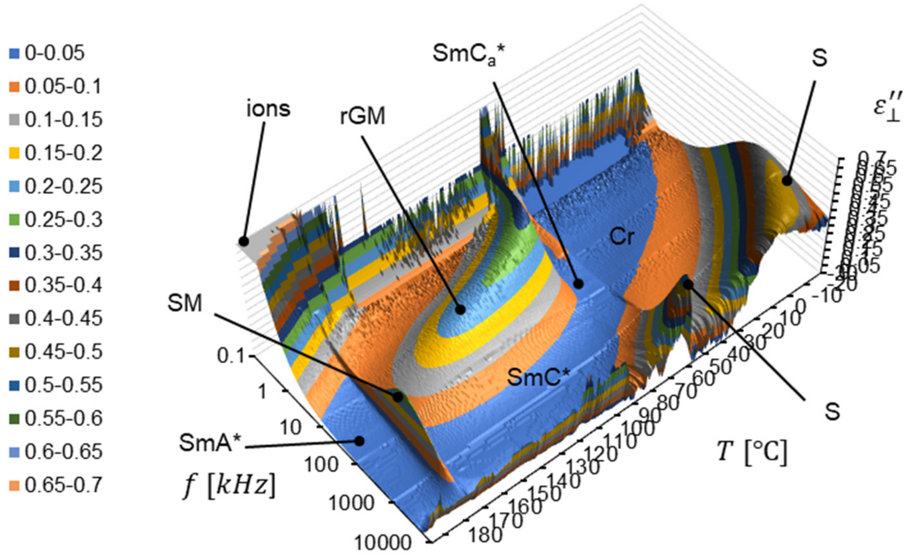

Figure S9. Imaginary part $\varepsilon''_{\perp}$ of dielectric permittivity for the compound 7PhPhCH2O. In 5 μm thin cell measurement at cooling, planarly aligned, with gold electrodes. Cooling rate 0.5°C/min. The 10 V DC field. The SmA* (soft mode: SM), the SmC* (residual Goldstone mode: rGM), the SmCa* (molecular S-mode) phases, and molecular crystal (Cr) (molecular S-mode) are observed.

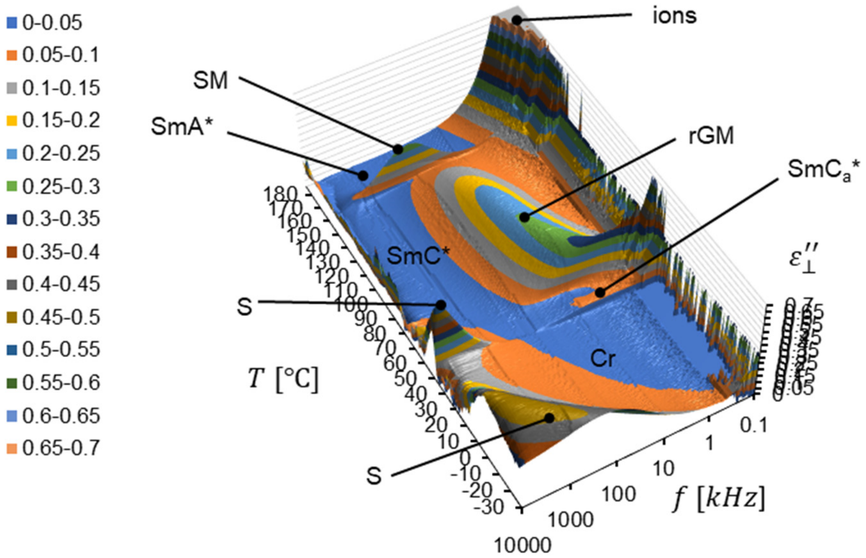

Figure S10. Imaginary part $\varepsilon''_{\perp}$ of dielectric permittivity for the compound 7PhPhCH2O. In 5 μm thin cell measurement at cooling, planarly aligned, with gold electrodes. Cooling rate 0.5°C/min. The 10 V DC field. The SmA* (soft mode: SM), the SmC* (residual Goldstone mode: rGM), the SmC_a* (molecular S-mode) phases, and molecular crystal (Cr) (molecular S-mode) are observed.
